# Progress on Ras/MAPK Signaling Research and Targeting in Blood and Solid Cancers

**DOI:** 10.3390/cancers13205059

**Published:** 2021-10-10

**Authors:** Martha Dillon, Antonio Lopez, Edward Lin, Dominic Sales, Ron Perets, Pooja Jain

**Affiliations:** Department of Microbiology and Immunology, Drexel University College of Medicine, Philadelphia, PA 19129, USA; mad452@drexel.edu (M.D.); arl345@drexel.edu (A.L.); ewl35@drexel.edu (E.L.); djs487@drexel.edu (D.S.); rp847@drexel.edu (R.P.)

**Keywords:** Ras signaling, leukemia, ATLL, MEK, ERK, viral oncogenesis, solid tumors

## Abstract

**Simple Summary:**

The Ras-Raf-MEK-ERK signaling pathway is responsible for regulating cell proliferation, differentiation, and survival. Overexpression and overactivation of members within the signaling cascade have been observed in many solid and blood cancers. Research often focuses on targeting the pathway to disrupt cancer initiation and progression. We aimed to provide an overview of the pathway’s physiologic role and regulation, interactions with other pathways involved in cancer development, and mutations that lead to malignancy. Several blood and solid cancers are analyzed to illustrate the impact of the pathway’s dysregulation, stemming from mutation or viral induction. Finally, we summarized different approaches to targeting the pathway and the associated novel treatments being researched or having recently achieved approval.

**Abstract:**

The mitogen-activated protein kinase (MAPK) pathway, consisting of the Ras-Raf-MEK-ERK signaling cascade, regulates genes that control cellular development, differentiation, proliferation, and apoptosis. Within the cascade, multiple isoforms of Ras and Raf each display differences in functionality, efficiency, and, critically, oncogenic potential. According to the NCI, over 30% of all human cancers are driven by *Ras* genes. This dysfunctional signaling is implicated in a wide variety of leukemias and solid tumors, both with and without viral etiology. Due to the strong evidence of Ras-Raf involvement in tumorigenesis, many have attempted to target the cascade to treat these malignancies. Decades of unsuccessful experimentation had deemed Ras undruggable, but recently, the approval of Sotorasib as the first ever KRas inhibitor represents a monumental breakthrough. This advancement is not without novel challenges. As a G12C mutant-specific drug, it also represents the issue of drug target specificity within Ras pathway; not only do many drugs only affect single mutational profiles, with few pan-inhibitor exceptions, tumor genetic heterogeneity may give rise to drug-resistant profiles. Furthermore, significant challenges in targeting downstream Raf, especially the BRaf isoform, lie in the paradoxical activation of wild-type BRaf by BRaf mutant inhibitors. This literature review will delineate the mechanisms of Ras signaling in the MAPK pathway and its possible oncogenic mutations, illustrate how specific mutations affect the pathogenesis of specific cancers, and compare available and in-development treatments targeting the Ras pathway.

## 1. Introduction

**Figure 1:**
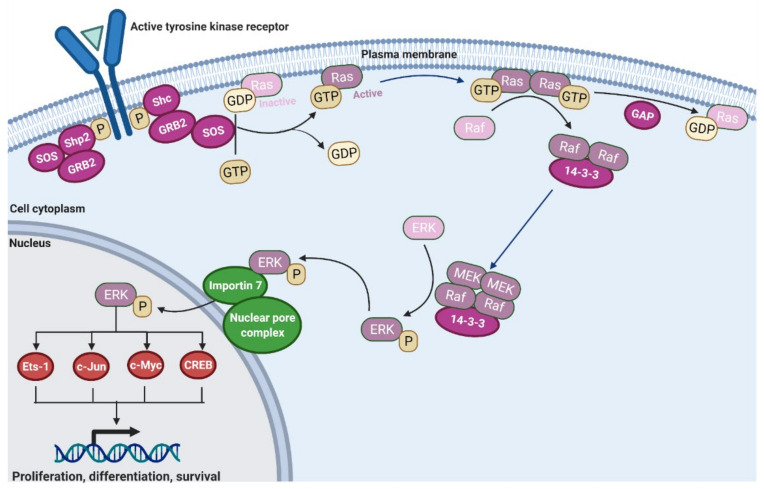
The MAPK cascade. Once a ligand binds the tyrosine kinase receptor, it self-phosphorylates [18]. This creates binding sites for Shc and Shp2. GRB2 can associate with either and then recruit SOS [19,20]. SOS is a guanine exchange factor for Ras and induces the exchange of GDP for GTP [21]. Now active Ras will dimerize and bind Raf [21]. After activating Raf, GTPase activating proteins (GAP) will hydrolyze the GTP to GDP to return Ras to its resting inactive state [22]. The active Raf dimers will recruit MEK [23], which then activates ERK [3]. ERK interacts with Importin 7 at the nuclear envelope to facilitate its entry through the nuclear pore complex into the nucleus [24,25]. Once inside, it phosphorylates multiple transcription factors to alter gene expression in the cell and induce proliferation and survival [26].

The MAPK cascade is a vital cellular network, which regulates apoptosis, development, differentiation, and proliferation (Reviewed in [1,2]). The network causes cellular changes through modulating gene expression. It achieves these alterations by integrating signaling induced receptor-ligand interactions, phosphorylation cascades, and modulation of transcription factor activities. Much progress has been made in understanding the role of MAPK signaling in cancer, which has resulted in the development of targeted therapies aimed at curbing aberrant MAPK signaling [3]. There are three main cascades in the mammalian MAPK family: classical MAPK (ERK), C-Jun N-terminal kinase (JNK), and p38 kinase [4]. The classical MAPK family involves the Ras-Raf-MEK-ERK cascade of proteins [5]. Many cancers have mutations in the classical MAPK cascade that contribute to unregulated cellular division. Studying the pathway’s normal physiology has allowed therapies to be developed that are able to treat malignancies by differential targeting of proteins implicated in the MAPK/ERK cascade (Figure 1). Notably, Ras, the master regulator of the MAPK pathway, is inherently associated with the development of cancer. This has recently (2020) been described in detail [6]. Once thought to be untargetable, recent breakthroughs in allele-specific covalent inhibitors have opened new doors for cancer therapy [7]. Downstream from Ras, all Raf isoforms enhance the catalytic activity of Mitogen activated protein kinase/ERK kinase (MEK1) but have differing efficacy. BRaf is generally shown to be the most effective at inducing MEK activation, and ARaf is shown to be the least effective [8,9]. However, in targeting BRaf, scientists have found the challenge of paradoxical activation and aberrant signaling independent of Ras activation [10]. Additionally, p21-activated kinases 1 (PAK1) phosphorylate MEK1 to increase association with Raf proteins, allowing c-Jun N-terminal kinase/stress-activated protein kinase (JNK/SAPK) cross-pathway enhancement of the MAPK cascade [11]. MEK1 activates its sole downstream target, extracellular-signal-regulated kinases 1,2 (ERK1/2) by phosphorylation of threonine and tyrosine residues [12]. ERK has over 200 targets within the cell that contribute to proliferation, differentiation, cell survival, and other diverse cellular processes. The length and degree of ERK signaling plays an important role within the cell. Sustained moderate ERK signaling over many hours downregulates anti-proliferative genes which prevent cell-cycle progression from G0/G1 into S phase. This allows the expression of cellular signals to promote progression including cyclin D1 [13,14,15]. In contrast, transient higher levels of ERK signaling induce CDK-inhibitor protein expression, including p21 and p27, halting the cell-cycle progression [15,16,17].

Another important process that contributes to malignancy is viral oncogenesis, which is believed to comprise 12% of all clinically observed cancers and involves similar dysregulation of these conserved growth and signaling pathways [27]. There are several viruses known to be oncogenic in humans, including Epstein–Barr virus (EBV), hepatitis B and C viruses (HBV/HCV), human T-cell leukemia virus type 1 (HTLV-1), and human papillomaviruses (HPV). Although distinct, these viruses share the similar characteristic of modulating host cellular processes to proliferate and propagate themselves or to avoid immune detection and clearance from the body [28,29,30]. We have been investigating HTLV-1 infection, pathology, and associated adult T-cell leukemia/lymphoma (ATLL) [31,32,33,34,35,36,37]. 

Targeting the Ras signaling pathway has been a longstanding challenge, frustrating the efforts of scientists for decades with the Ras protein itself even being considered undruggable [7]. However, recent breakthroughs in MAPK/ERK pathway inhibitors have led to the approval of a novel, mutation-specific drug that directly inhibits Ras. Sotorasib is a first of its kind KRas G12C mutant targeting anti-cancer therapy and is currently approved for the treatment of non-small cell lung cancer (NSCLC) (Clinical Trial NCT03600883). Meanwhile, Sorafenib, the first FDA approved drug to treat HCC, is one of the only approved targeted drug therapies for advanced HCC, targeting Raf. However, challenges remain in the specificity of drugs to mutational profiles. Resistance easily arises due to tumor genetic heterogeneity, exacerbated by a lack of pan-Ras noncovalent inhibitors, and other factors [38,39,40,41]. Downstream, the challenge of paradoxical activation of the pathway by Ras-Raf inhibitors must also be addressed [10]. In this review, we have examined the role Ras-Raf signaling plays in the pathology of leukemias/lymphomas and solid tumors by compiling the current understanding of this signaling network in various cancers. The role of Ras-Raf signaling will also be discussed in the context of currently available therapies and ongoing clinical trials.

## 2. Activation of Ras and Raf Proteins

**Figure 2:**
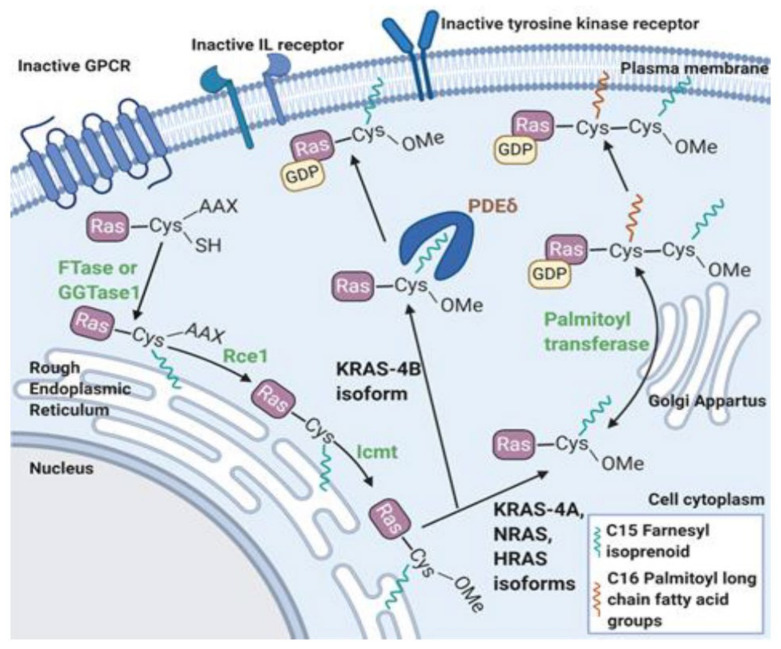
Post translational farnesylation of Ras protein. The first modification is prenylation, preferentially performed by farnesyl transferase (FTase) [44]. This is initiated after recognition of the CaaX box on Ras’s C-terminus by FTase. Alternatively, the KRas-4B and NRas isoforms can be acted on by geranylgeranyl transferase 1 (GGTase1) if FTase is inhibited [43,45]. The farnesyl and geranylgeranyl moieties add enough hydrophobicity to enable Ras insertion into the endoplasmic reticulum membrane. Ras converting enzyme (Rce1) performs a final cleavage of the CaaX residues before isoprenylcysteine carboxyl methyltransferase (Icmt) adds a carboxymethyl group [44]. The final processing and transfer to the plasma membrane is isoform specific. Due to the farnesyl tail and a five amino acid sequence motif (Lys- Ser- Lys-Thr-Lys) in the C-terminus region, KRas-4B is directly chaperoned to the membrane by phosphodiesterase delta (PDEδ) [43,46,47]. Lacking the necessary motif, all other isoforms enter the Golgi apparatus for reversible palmitoylation by palmitoyl transferase. From the Golgi apparatus, HRas and NRas are trafficked to the plasma membrane on motile vesicles [48]. KRas-4A is trafficked by a poorly understood Golgi- independent route depending on mitochondrial function and class C vacuolar protein sorting (vps) proteins [49]. Afterwards, all isoforms associate with the membrane through their respective two-point anchors: the farnesyl modification and polybasic region of six lysines for KRas4b and the palmitoyl and farnesyl modifications for the other isoforms [50].

The master regulator of the classical MAPK cascade is the Ras protein, which is encoded by three genes, *NRas*, *HRas*, and *KRas*. These genes produce four active isoforms sharing a highly conserved structure and a unique C-terminal hypervariable region. These distinct C-terminal variations result in different post translational modifications that create different Ras isoforms with distinct efficacy, cellular distribution and functionality [42]. To localize to the plasma membrane where it can recruit Raf, all four Ras isoforms require post-translational modification after synthesis. A tetrapeptide signal at the carboxyl terminus, the CaaX box, serves as the motif recognized by farnesyl transferase (FTase) to initiate these changes (Figure 2) [43,44]. Once anchored onto the plasma membrane, signaling through Ras can be induced by via cytokine receptors, tyrosine kinase receptors, and G-protein-coupled receptors (GPCRs).

### 2.1. Ras Activation by the Tyrosine Kinase, Interleukin (IL) Receptors, and G-Protein Coupled Receptors

Tyrosine kinase and interleukin receptors utilize the same mechanism to interact with the Ras cascade. After cytokine binding, subsequent receptor dimerization allows transphosphorylation by the clustering Janus kinase 2 (JAK2) proteins bound to both β chains [51]. At high cytokine concentrations, adaptor protein Shc’s src homology 2 domain (SH2) binds to these phosphorylated tyrosine residues. While it lacks inherent catalytic function, Shc serves as a phosphorylated anchor for growth factor receptor bound protein 2 (Grb2) to bind. Grb2 then associates with son of sevenless (SOS), a guanine nucleotide exchange factor (GEF) for Ras [51,52,53,54].

**Figure 3:**
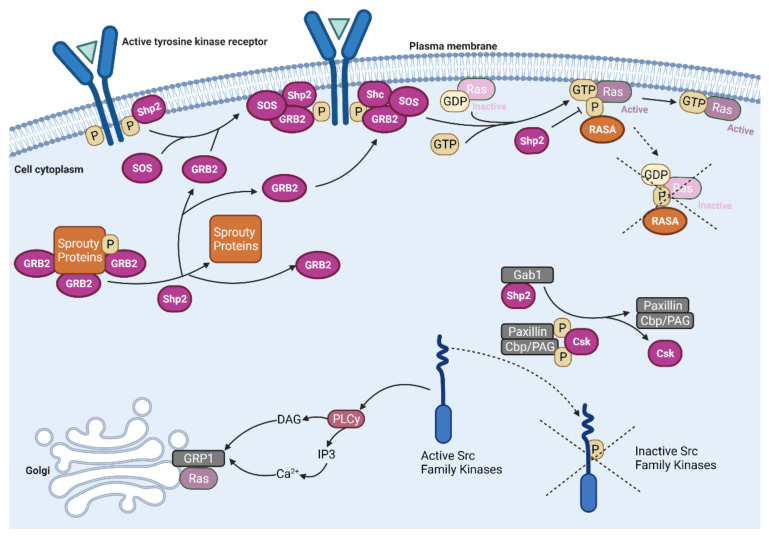
The roles of src homology region 2 domain-containing phosphatase 2 (Shp2) in the Ras-Raf-MEK-ERK pathway. Shp2 is a GRB2 scaffolding protein that anchors it to tyrosine kinase receptors [56]. Additionally, Shp2 dephosphorylates Sprouty family proteins to release sequestered GRB2 molecules [57,58]. It also dephosphorylates Ras docking sites of RASA, a Ras GTPase activating protein (Ras-GAP) that accelerates the hydrolysis of Ras bound GTP [59,60]. This allows more active Ras-GTP molecules to accumulate instead of being converted to inactive Ras-GDP. After being recruited by Gab1, Shp2 can also dephosphorylate the binding sites of Paxillin and the Csk binding protein/phosphoprotein associated with glycosphingolipid-enriched microdomains (Cbp/PAG). This prevents the docking of C-terminal Src kinase (Csk) on Src family kinases (SFKs) and inhibits its activity by phosphorylating Tyr527 [61,62,63,64]. When active, SFKs initiate a signaling pathway through phospholipase C γ (PLCγ), diacylglycerol (DAG) and calcium. This results in the recruitment of the Ras guanine nucleotide exchange factor RasGRP1, directing it to the Golgi to activate intracellular Ras molecules [65,66].

Grb2 can also associate with src homology region 2 domain-containing phosphatase 2′s (Shp2) [55] (Figure 3). This protein acts as a scaffolding protein, serving as a link to tyrosine kinase receptors via its two SH2 domains and Grb2 at its C terminus tail. It also has protein tyrosine phosphatase domain, which inactivates several negative regulators of the MAPK pathway [56]. Shp2′s dephosphorylation releases additional Grb2 molecules from sequestration by Sprouty family 1–2 proteins [57,58]. It also dephosphorylates Ras’ docking sites of RASA, a Ras GTPase activating protein (Ras-GAP) that accelerates the hydrolysis of Ras’ bound GTP. By preventing RASA’s binding, Ras-GTP accumulates and propagates its effects longer [59,60]. Shp2 also indirectly affects Ras’s signaling through the Src family kinases (SFK), cytosolic protein tyrosine kinases that play roles in cell proliferation and survival. Src proteins have two tyrosine sites that play a role in regulation. Auto dephosphorylation of Tyr416 contributes to Src activation, while Tyr527 phosphorylation by C-terminal Src kinase (Csk) is inhibitory [61]. Before it can inhibit SFKs, Csk activity is determined by the docking protein Paxillin and the Csk binding protein (Cbp). CBP is also known as the phosphoprotein associated with glycosphingolipid-enriched microdomains (PAG). When Shp2 is recruited by Gab1, it prevents the docking of Csk by dephosphorylating paxillin and Cbp/PAG. Csk molecules then dissociate from SFKs and allow them to propagate ERK signaling [62,63,64].

At basal signaling levels, Ras proteins are bound to GDP and impeded from interacting with its effectors. When SOS is activated, it facilitates Ras’ exchange of GDP for GTP, resulting in an activated Ras confirmation. [43,46,67]. While SOS is ubiquitously expressed throughout the body to act downstream as part of receptor coupling, there are two additional guanine exchange factors (GEFs) that are tissue specific and facilitate the activation process. Both additional GEFs are distributed in the central nervous system [68,69]. Ras protein-specific guanine nucleotide releasing factor 1 (Ras-GRF1) is also expressed in the pancreas [70], while Ras-GRF 2 is additionally expressed in T-Cells [71,72].

While Ras has some slow intrinsic GTPase ability, its signal is usually terminated in conjunction with GTPase activating proteins [22,73]. Ras GAPs catalyze the hydrolysis of the bound GTP, accelerating the process by a factor of 10^5^ [74,75]. By ending the signaling driving the MAPK pathway, these tumor suppressors prevent unlimited and unregulated cell proliferation and other cellular outcomes downstream of Ras [76,77]. Some of the most studied members of this family include neurofibromin and DAP2IP [76]. Whether GAP’s loss of function occurs through germline or somatic mutations [78], proteasomal degradation [79] or epigenetic silencing [80,81], the resulting proliferation of cells by prolonged Ras signaling are recognized in having a role in many cancers including lung [82], prostate [83,84], and hepatocellular cancers [85,86].

Ras molecules have also been shown to cluster together on different microdomains of the plasma membrane. Inactivated HRas-GDP isoforms have an affinity for lipid rafts on the plasma membrane near its triggering receptors and GEFs [67,87]. Lipid rafts are transient nanoscale clusters of protein and cholesterol within the plasma membranes. These liquid-ordered regions form around the HRas isoforms, partly due to the deep insertion of the palmitoyl moieties into the bilayer resulting from the GDP-induced confirmation [88]. Once GDP is exchanged for GTP, activated HRas’ conformational change induces changes in the N terminal catalytic domain and the hypervariable linker domain [89]. These changes decrease the extension of the palmitoyl moiety and release HRas-GTP from the liquid-ordered region. HRas-GTP enters a new liquid-disordered microdomain which allows for preferential interaction with the scaffolding protein Galectin-1 (Gal-1) as well as interaction with Raf and other subsequent signaling proteins [88,90]. Gal-1 is a known regulator of Ras nanoclustering specific to HRas. When Ras nanoclusters begin to recruit effector proteins, it induces Raf dimerization. Gal-1 binds to the Ras-binding domain on two Raf molecules, stabilizing the Raf dimer and conveying stability of Ras dimers and the whole nanocluster. While Gal-1 is specific to H-Ras, other scaffolds for the other isoforms include galectin-3, nucleophosmin and caveolae [21]. Due to the lack of palmitoylation motifs, KRas proteins have their own nonoverlapping, cholesterol-independent liquid-disordered microdomains. These clusters, like KRas signaling, are actin dependent [87]. NRas localizes to the borders of liquid ordered/liquid disordered microdomains as the GTP bound state preferentially localizes to cholesterol sensitive clusters [88,91]. These patterns of clustering and microdomain association have been a recent interest of research, as these patterns are suggested to play a role in determining how each isoform has a distinct function and signaling throughout the body. Where they cluster facilitates interactions with their activators, scaffolds and substrates, as well as with other Ras molecules to promote the dimerization that supports coupling with Raf. It can also determine the susceptibility of each isoform to different modifications. Differences in the microenvironments has been shown to determine that HRas and NRas can be targeted for ubiquitination signaling, allowing them to be transported to endosomes’ signaling network [92]. As the microdomains of Ras are better mapped and understood, they will provide more understanding on the regulation of Ras and how mutations in distinct isoforms may affect tissues differently.

**Figure 4:**
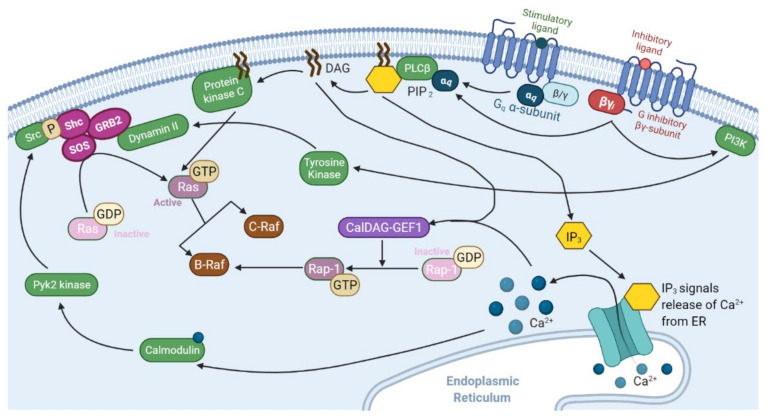
G protein-coupled receptor subunits Gα_q_ and Gβγ_i_ interaction with the Ras-Raf pathway. G-proteins are heterotrimeric guanine nucleotide binding proteins with α-, β-, and γ-subunits. When a ligand binds to the extracellular portion of the receptor, it confers a guanine nucleotide exchange factor confirmation that induces the α-subunit to exchange its bound GDP to GTP. This causes the α-subunit to disassociate from the receptor and βγ- subunit. Both the α and βγ subunits effect changes in the cell, before the α subunit hydrolyzes the GTP and returns the receptor complex to its inactive state [94,95,107,108]. Both α_q_ and βγ_i_ activate phospholipase C-β (PLCβ) to create the second messengers of diacylglycerol (DAG) and inositol-1,4,5-triphosphate (IP3) through the hydrolyzation of phosphatidylinositol 4,5-bisphosphate (PIP2) [109]. DAG activates PKC, which directly phosphorylates and activates Ras proteins [110,111]. IP3 stimulates the calmodulin pathway and Pyk2 kinase by way of inducing calcium release from the endoplasmic reticulum [112]. The resulting phosphorylation provides the base for Shc anchoring and recruitment of Ras’ guanine exchange factor complex [113]. Both DAG and IP3 play a role in allosterically controlling CalDAG-GEF1, a guanine exchange factor for Rap1 [94,114]. Once Rap1 has exchanged its bound GDP for GTP, it can activate BRaf in the place of Ras [115]. Furthermore, βγ_i_ activates phosphoinositide 3-kinase (PI3K), which augments cell signaling from tyrosine kinase receptors to increase Dynamin II [116], an additional anchor for Shc and Ras’ GEF complex [117,118,119].

**Figure 5:**
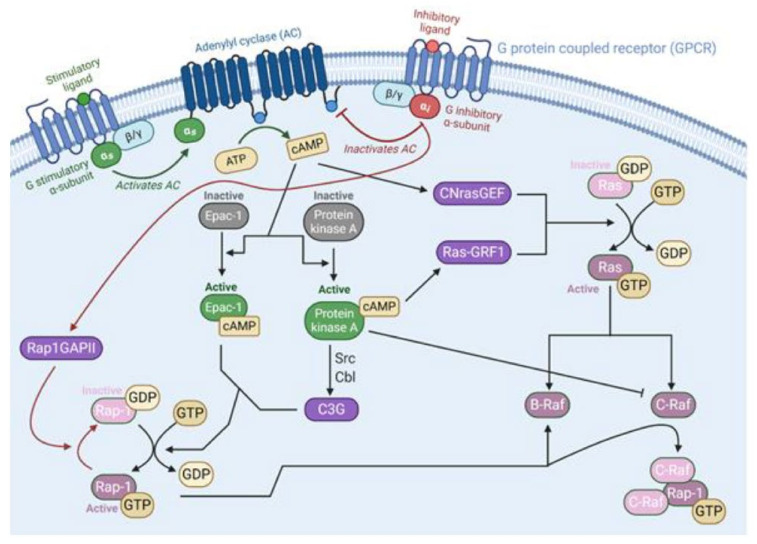
G-protein-coupled receptor subunits Gαs and Gαi interaction with the Ras-Raf pathway. When activated by a ligand, the GPCR is induced to exchange its bound GDP for GTP, freeing the α subunit to act on the cell. α_s_ acts on adenylyl cyclase (AC) to increase the production of the second messenger cAMP from ATP [120]. In turn, cAMP activates Epac-1 [121] and protein kinase A [120]. Epac-1 and C3G, a downstream molecule from PKA, are guanine-nucleotide exchange factors for Rap-1 and induce the exchange of GDP to GTP to activate it [121,122]. Both activate Rap-1, which can modulate the activity of BRaf [115]. Protein Kinase A has other mechanisms of action as well. It can directly prevent the activation of CRaf through phosphorylation. Both activate Rap-1, which can modulate the activity of BRaf [115]. Protein kinase A has other mechanisms of action as well. It can directly prevent the activation of CRaf through phosphorylation [123]. It also activates a Ras GEF, RasGRF1, to start the MAPK cascade in certain cells [124]. In contrast, αi inhibits adenylyl cyclase and produces opposite effects. It also activates a Ras GEF, RasGRF1, to start the MAPK cascade in certain cells [124]. In contrast, αi inhibits adenylyl cyclase and produces opposite effects.

Recently, there has been a renewed interest in mapping the pathways of G-protein coupled receptors (GPCRs) and their roles in tumorigenesis. Many neuropeptides, including galanin, neurotensin, and gastrin-releasing peptide, have been found to stimulate proliferation and survival of small cell lung carcinoma cells through GPCR activation of ERK activity [93]. Each peptide signals through their specific GPCR, and several GPCR groups interact directly with the Ras-Raf-ERK-MAPK pathway as illustrated in Figure 4 and Figure 5. In various cell types, the effects differ depending which α-subunit isoform is utilized. Gα_s_ induces adenylyl cyclase to produce the second messenger cAMP, triggering both protein kinase A (PKA) and exchange protein directly activated by cAMP (Epac-1). Downstream, this can modulate the activity of Raf proteins: increased BRaf activity or inhibition of c-Raf action [94,95,96]. In adrenal medullary cells, Gα_s_ signals through cAMP, PKA and Rap-1 potentiates the effects of growth factors and supports differentiation into sympathetic neurons [97]. In comparison, Gα_i_ subunits have the opposite effect by deactivating adenylyl cyclase and decreasing the amount of cAMP present [94]. Winitz et al. showcased G_i_ activation of CRaf-MEK-ERK in fibroblasts with the acetylcholine muscarinic m2 receptor [98] (Figure 5). Additionally, the βγ_i_ subunits of these receptors, as well as the G_q_ GPCR family, can have additional effects in some cells through the activation of phospholipase C-β (PLCβ), which will lead to the activation of the Raf-MEK-ERK pathway to induce chemotaxis and proliferation. Della Rocca et al. found that stimulation of α1B or α2A adrenergic receptors triggered these pathways to cause a rapid 5–10-fold increase in ERK phosphorylation. This activation relies on the phospholipase C, calmodulin, Pyk2 and Src pathways and suggested that fibroblasts, ovary cells and neuroblastoma cells used this mechanism for cellular proliferation [99]. Βγ subunits have also been shown to cause the accumulation of GTP-bound Ras proteins, prolonging their signals [100]. Furthermore, constitutively active G_q_ receptor’s Gα_14_ subunits have been shown to increase the formation of GTP-bound Ras and the downstream phosphorylation of ERK in hepatocellular carcinoma cells [101]. G protein-coupled receptors are also part of chemokine and environment-sensing axes that are being researched as possible drug targets in B-cell lymphomas as well as prostate and ovarian cancers [95,102,103,104,105]. In addition to direct activation of the Ras-Raf pathway, some GPCRs have also been shown to mediate and increase the interaction between the scaffolding protein 14-3-3 and CRaf to increase CRaf signaling [106].

### 2.2. Activation and Regulation of Raf Protein

The classical MAPK cascade continues with the activation of the serine/threonine kinase Raf. There are three Raf isoforms that can be activated: ARaf, BRaf, and CRaf (also called Raf-1). All three isoforms share a similar conserved two-lobe structure connected by an oscillating hinge. These structures can be further broken down into the Ras-binding domain (RBD), the cysteine-rich domain (CRD) and an acidic N-terminus (NTA) on the N-terminal side as well as a serine/threonine-kinase domain and 14-3-3 binding motif on the C-terminal side. The intervening hinge region has a second conserved region (CR2) with another 14-3-3 recognition site [125,126]. The CRD domain is a conserved C1 domain, or a small domain found in many proteins that are activated at the membrane [126]. Despite this similarity, each isoform has varying activity levels and roles throughout the cell.

Before activation, or in non-dividing cells, Raf’s N-terminus docks onto and auto-inhibits the kinase region to prevent any activity [127]. This autoinhibition is stabilized by the regulatory 14-3-3 proteins [126]. These are phosphoserine/phosphothreonine binding proteins with two Raf binding sites. In the inactive state, 14-3-3 binds to both the C-terminus and the CR2 site in the intervening hinge motifs of a single Raf molecule. [128,129,130,131]. To facilitate 14-3-3 binding, the CR2 site is phosphorylation by several different enzymes, including protein kinase A (PKA) [23,132], AKT [133,134], AMPK [135,136,137] serum/glucocorticoid regulated kinase (SGK) [128] and LATS1 [138]. All parts of this inactivating bundle are oriented by the CRD domain at the center of the complex. This positions active site of the C-terminal kinase domain to point outwards from the bundle and coordinate with Raf’s substrate, MEK. Both Raf and MEK remain inactive as their alpha helical domains are still displaced, but have their active sites aligned around an ADP molecule [126]. Additionally, the CRD domain is prevented from interacting with the cellular membrane [139].

To switch confirmations, the Raf protein must first be recruited to the plasma membrane. An anchored and activated Ras will bind to the RBD of Raf with nanomolar affinity due to hydrogen bonding and electrostatic interactions to form an extended β sheet structure. There is an inherent flexibility around this binding, allowing the Raf molecules to rotate to enter better positions. After this binding, the CRD domain is released from the autoinhibition complex. A zinc finger motif forms between the RBD, the CRD and the short five amino acid linker region between them, allowing the two domains to interact directly with each other and as one extended structure [139,140]. The CRD domain will then also creates a large hydrophobic interface with Ras to stabilize its interaction with Raf. The hydrophobic portions of CRD will also contact the bilayer’s phospholipids to anchor the complex with the membrane [139]. Furthermore, the release of CRD exposes the phosphorylated serine regulatory site in the CR2 region of Raf. Protein phosphatase 1 (PP1) and the leucine rich repeat scaffold protein SHOC2 removes the phosphorylation, disrupting the inhibitory interaction with 14-3-3 [141,142,143,144,145]. This dichotomy between the phosphorylation of the CR2 site acts as a valuable regulation point for Raf activity and allows many other pathways throughout the cell to feedback and influence Raf regulation. Further stabilization is due to the Ras clustering. Clustering close together allows nearby Raf molecules to dimerize [146]. The dimerization allows the autophosphorylation of the pair’s NTA motifs and activation loop segment on the C-lobe [146,147]. This is further supported when 14-3-3 binds to the C-terminal phosphoserine sites (Ser^621^ on Raf-1 and Ser^729^ on B-Raf) of both Raf proteins to strengthen the dimerization and promote Raf activity.

### 2.3. Ras-Raf-MEK-ERK Pathway Interaction with p53

In addition to the transcription factors that ERK targets, the Ras-Raf-MEK-ERK pathway interacts with several other key cellular pathways to mediate mitogenic signaling. To keep cells dividing, the activation of the classical MAPK cascade prevents the acetylation of p53′s DNA binding domain. Without this acetylation, p53 has decreased transcriptional activation of cyclin-dependent kinase inhibitor p21, effectively blocking the p53/p21 axis that would induce cell cycle arrest [148,149]. Additionally, inactivation of the p53/p21 cascade can induce cell division through the Raf-MEK-ERK cascade independent of Ras activation [148,149]. When Drosten et al. knocked down expression of p53 or p21 in Rasless cells, they observed proliferation and an activation of the Raf-MEK-ERK signaling pathway, as seen by increased phosphorylation of MEK and ERK. However, proliferation was not seen in cells lacking Raf, MEK or ERK. They therefore hypothesized that there was a p53-dependent feedback loop that decreased the activity of the Raf-MEK-ERK pathway [148]. In tumorigenesis, this inactivation could be due to lost p53 expression, a common mutagenic step in many cancers. Drosten’s hypothesis suggests that these common mutations remove negative feedback on cellular proliferation signals induced by the Raf-ERK-MEK pathway. This could explain some tumors’ continued proliferation despite DNA damage [150]. Furthermore, activating mutations of ERK can lead to cisplatin resistance in cancer when paired with inactivating p53 mutations [151]. This pair of mutations also often coexists in pancreatic cancer [152] and colon cancer [153].

Other research suggests that the two cascades interact through human double minute-2 protein (Hdm2), which inhibits p53 by sequestration to repress its transcriptional activity. Hdm2 also acts as an E3 ubiquitin ligase to promote nuclear export and degradation of p53 [154]. Once activated by ERK, Ets transcription factors bind to the promoter region of Hdm2, increasing its expression and ultimately causing the degradation of p53. In many cancers, p53 function is lost due to inactivating mutations, an overexpression of Hdm2 [153] or a decrease in p14ARF antagonization of Hdm2′s ubiquitin ligase activity [155]. In some tumor development, Ras mutations may increase Hdm2 levels enough to block p53 from inducing apoptosis or arresting growth in the face of DNA damage. This could induce the radiation resistance found in some tumors [153]. Physiologically, the classical MAPK pathway can be activated by hormones, growth factors, and differentiation factors. In malignant cells, additional activation can be due to aberrant function resulting from chromosomal abnormalities, genetic mutations, overexpression of upstream receptors, or innate mutations of the Ras-Raf-MEK-ERK pathway proteins themselves [156].

## 3. Ras-Raf Pathway Mutations

A major source of dysregulation for the MAPK-ERK pathway is the mutations affecting the proteins of the pathway. These mutations generally affect Ras and Raf and result in regulatory dysfunction that contributes to oncogenesis [157,158]. Therefore, it is important to discuss the frequency of these mutations and how they contribute to abnormal signaling.

### 3.1. Ras Mutations

Ras mutations are present in between 15 and 30% of cancers [159,160] and often result in pathway hyperactivation [161]. The frequency of these mutations and the location of each mutation are specific to each type of cancer [157,161]. Blood cancers commonly have NRas and KRas mutations (Table 1), whereas the majority of solid organ tumors with Ras mutations (Table 2), including colorectal and pancreatic cancers, generally only have KRas mutations. These KRas mutations are found at much higher rates than Ras mutations in blood cancer overall [160]. Depending on the Ras isoform subtype, there are hotspots for mutation that are unique: KRas is most commonly G12 mutated, and NRas and HRas are most commonly Q61 mutated, although HRas has an abundance of G12 and G13 mutations as well [160]. Mutations in these regions are oncogenic because they disrupt interactions between the Ras protein and GTP-ase activating proteins (GAPs) [22]. GAPs promote GTP hydrolysis and therefore functionally inactivate Ras. Without GAP-catalyzed GTP hydrolysis, Ras can remain active in the absence of an upstream signal and can contribute to oncogenesis. Loss of GAP function has been associated with neurofibromatosis and a series of cancers, including lung, hepatocellular, and prostate cancers [22,76,162].

The type of oncogenic cell expressing the Ras mutant can also drive mutations on the MAPK pathway. Some Ras mutants transform healthy cells more aggressively into malignant cells or to appear at earlier stages of malignancy [212]. Complicating the situation further, the missense mutations in the Ras codon hotspots have also been shown previously to result in different interactions with other proteins [157,213]. With many variables affecting how a specific Ras mutant behaves, researchers have found that response to pharmacological intervention can depend on the mutation subtype and currently the data suggest that new forms of treating cancer via Ras targeting may require distinct treatment methods [212]. As for how these mutations can be induced, Ras oncogenes have been known for decades to occur in rats treated with carcinogens [214]. The type of mutagen introduced has been shown to induce varying Ras mutant subtypes [157]. The type of mutagen introduced has been shown to induce varying Ras mutant subtypes [157]. Tissue exposure to mutagens drives some of the differences in mutational frequency seen in Ras mutant subtypes. This exposure does not explain why a tumor of a tissue may have Ras isoforms with different mutant subtypes (e.g., a tumor with HRas favoring 61 codon mutations but KRas 12 codon mutations). Work has been done showing that local tissue signaling networks may be responsible for a particular kind of *Ras* mutation and that certain co-mutation events involving KRas play a role in the specific location of the KRas mutation [215]. Ras isoforms have been shown to differ in DNA damage but not repair rates, with codon 12 binding better to carcinogens on KRas compared with HRas and NRas [157,216]. In addition to increased binding of carcinogens, KRas was shown to be more at risk for damage from UV radiation than NRas. This is despite NRas having a high frequency of mutation in malignant melanoma, a cancer associated with UV light exposure, and is due to the higher prevalence of thymine–thymine sites in the KRas gene [216]. KRas’s higher susceptibility to chemical and UV mutagenesis may explain why it is more frequently seen in cancers, although more work is needed to understand why certain codons may be preferentially mutated in Ras isoforms.

### 3.2. Raf Mutations

Raf mutations affecting the serine/threonine kinase contribute to a series of development disorders and are thought to contribute to approximately 8% of cancers [158,217]. Hundreds of missense mutations have been found affecting the Raf gene that result in various degrees of hyperactivation and downstream activation of ERK, resulting in oncogenesis [188]. Of the three Raf isoforms, ARaf, BRaf, and CRaf, BRaf is the isoform more commonly mutated in cancer [217]. The location of the mutation on BRaf has a large effect on its ability to increase activity several fold over wild type, as mutations in the activation loop and phosphate binding loops impair their inhibitory interactions leading to an active conformation of the Raf protein [217,218]. The V600 location on BRaf is the most important location for mutations on this protein, with mutations occurring here in 92% of oncogenic forms of BRaf [218]. Basal kinase activities as high as 480-fold occur depending on the mutant form with V600D, V600E, V600K, and other similar mutants having some of the highest basal kinase and phosphorylated ERK levels [187,218]. Alternate mutations other than V600E at the same location were shown to have higher or similar kinase activity, however the glutamate replacement requires a single base substitution and is therefore seen at higher rates in cancers. Various mutants with locations outside of the 600 amino residues have been found that lower rates of kinase activity. It is thought that there may need to be a varied activation of the BRaf protein due to the fact that overactivation of ERK leads to senescence [188].

### 3.3. MEK and ERK Mutations

Mutations in MEK and ERK are less studied but have been noted in developmental disorders and in both naturally occurring neoplasms and in response to BRaf inhibitors as a mechanism for resistance when treating cancers such as melanoma [219,220,221,222]. ERK, although often overactive due to abnormal upstream activity, is rarely mutated in cancer [223]. MEK and ERK mutations are not nearly as common as Ras and Raf mutations, however they are vital to understanding mechanisms of resistance. This importance can be seen with the use of Raf inhibitors for cancers such as melanoma, which results in brief clinical improvement, but often ends with patients developing resistance through genetic and nongenetic processes [221]. Depending on the location of the mutation, MEK1 and MEK2 mutations have been shown to reduce BRaf and/or MEK inhibition by dabrafenib and trametinib, respectively [220,221,224]. ERK mutations that are clinically relevant have been seen in response to ERK and Raf inhibitors and are thought to mediate drug resistance [225,226]. Mutations were found that obstruct proper ERK inhibitor binding, resulting in ERK catalytic activity despite inhibitor treatment. ERK mutation mediated resistance was circumvented using MEK inhibition, suggesting that resistance due to mutations of ERK in response to various MAPK/ERK inhibitors may be superseded through combination therapy targeting multiple proteins in the pathway [225,226]. Combined inhibition using various MAPK/ERK inhibitors also superseded MEK and NRas mutations and therefore represents an avenue for cancer therapy [220]. Experimentally derived and naturally observed ERK mutations are reviewed in [223].

## 4. Ras-Raf Signaling in Various Types of Cancers

Aberrant signaling in the MAPK cascade holds significant weight in the development of lymphomas and solid tumors. Disruptions in signaling may be from a variety of causes not limited to genetic predispositions, as demonstrated in cases of virally associated cancers such as adult T-cell leukemia/lymphoma, Burkitt’s lymphoma, and hepatocellular carcinoma.

### 4.1. Leukemia/Lymphoma

Adult T-cell leukemia/lymphoma (ATLL) is an aggressive T-cell neoplasm characterized by the clonal expansion of lymphocytes, which displays monoclonal integration of the human T-cell leukemia virus type 1 (HTLV-1) provirus [227,228]. HTLV-1 was initially found in a patient cell line diagnosed with cutaneous T-cell lymphoma in 1979 and is known as the first human retrovirus to be discovered [229,230]. HTLV-1 proviral integration into the host chromosome is believed to drive many of the observed consequences of genomic instability and disruption of genome integrity [231,232]. The transformation of T-cells present in patients with ATLL is almost certainly dependent on the activities of Tax1 protein in infection/disease progression. However, it is suggested that Tax dependence is only required early during infection as Tax1 is rarely detected in the leukemic cells of ATL patients [228,231]. Tax1 is the HTLV-1 viral, trans-activator protein that has pleiotropic effects in activating and dysregulating cellular processes involved in growth and immunosurveillance and has been extensively studied regarding its activities as a tumor initiator in ATL [233,234]. Tax1 overexpression performed in cancer cell lines (breast, colon, and hepatoma) resulted in increased proliferation as quantified by MTT assay [235]. Additionally, protein analysis by Western blot demonstrated an increase in phosphorylation of all the members in the Ras-Raf pathway, suggesting Tax1-mediated augmentation of signaling in driving proliferation [235]. A crucial effect of Tax-mediated dysregulation is increased cellular proliferation through activation of MAPK. A whole-genome integration study conducted on a cohort of 370 ATL cases revealed numerous genomic alterations, such as activating mutations, gene fusions, and insertion/deletions [236]. These alterations overlap with genes that are targeted by and known to interact with Tax1 during HTLV-1 infection. A different study analyzing Ras signaling found that Tax1 expression resulted in increased Ras-GTP levels and increased phosphorylation of ERK, and that this correlated with an anti-apoptotic state. Apoptotic resistance was overcome when a Ras farnesylcystein mimetic (FTS, *S*-farnesylthiosalicyclic acid) was used in Tax 1-expressing cells, which also resulted in decreased levels of Ras-GTP and phosphorylated ERK, suggesting that the Ras-Raf-MEK-ERK signaling pathway partially contributes to apoptosis resistance in ATL [237].

Burkitt’s leukemia/lymphoma (BL) is a subtype of non-Hodgkin’s lymphoma (NHL) that is diagnosed as an aggressive neoplasm of B lymphocytes [238]. BL can be categorized into three types, endemic, sporadic, or immunodeficiency-related, and is characterized by a high rate of proliferation and apoptosis. For the endemic BL, infection or association with the Epstein–Barr Virus (EBV) is always observed, whereas it is only observed in 25–40% of cases of sporadic and immunodeficiency-related BL [239,240]. The defining feature of BL across all subtypes is the translocation of the oncogene *MYC* into proximity with either the immunoglobulin heavy or light chain, which results in continuous *MYC* expression that is believed to drive the high proliferation rate [241,242]. As such, the development of methods to target *MYC* has seen preclinical and clinical attention and represents an important future avenue to pursue in the treatment of BL [243,244]. The use of chemotherapy is effective in treating pediatric BL, however, caution is taken when treating adults due to the risk of tumor lysis syndrome [239]. Although the role of *MYC* in driving BL oncogenesis has been well characterized, the importance of other oncogenes such as those in the MAPK cascade is not as well researched. A study that analyzed the genomes of BL patients at primary diagnosis and at relapse detected mutations within the MAPK pathway, specifically in NRas [245]. While *MYC* mutations were also found in patients at primary diagnosis, the detection of NRas mutations at relapse suggests that dysregulation of MAPK can provide therapeutic resistance in patients undergoing chemotherapy.

### 4.2. Solid Tumors

Liver cancer was the fourth most common cause of cancer related death worldwide in 2020, with the vast majority being hepatocellular carcinoma (HCC) [246]. HCC is usually observed in patients with preexisting liver conditions, such as cirrhosis or chronic hepatitis B or C viral infections [247]. Common treatment modalities include surgical resection, liver transplantation, trans-arterial chemoembolization, radiation, ablation, and systemic therapies, including sorafenib [246,247]. This is an aggressive and lethal cancer often diagnosed at an advanced stage, and there is a need to develop additional therapeutics. HBV infection, the leading driver of HCC worldwide, induces mutations in tumor protein p53 and activation of oncogenic signaling [248]. The MAPK-ERK signaling pathway is highly active (50–100%) in most observed HCC cases, however, mutations of Ras or Raf genes are rarely observed in humans [206,249]. The lack of Ras-Raf mutations in HCC suggests that there is either improper upstream signaling or that there is a lapse in MAPK/ERK inhibition and regulation which results in overactive signaling.

The Ras-Raf pathway is known to play a critical role in HCC. Several members in the MAPK-ERK pathway are overexpressed in HCC. CRaf and MEK were found to be heavily upregulated in both cirrhosis and carcinoma, and CRaf was found to be heavily phosphorylated in nearly all cirrhosis and carcinoma samples tested by Hwang et al. [250]. Overexpression of Ras, MEK, and particularly CRaf were associated with worse prognostic outcomes [251]. The upregulation of Raf in HCC is consistent with previous work showing that it is key in tumor growth and angiogenesis in many different solid cancers and with data showing that Raf inhibition disrupts these two processes in HCC [251]. Additionally, a study comparing MEK1/2 levels between tumor and healthy HCC cells, found that only tumor cells showed high phosphorylated MEK1/2 levels and that an increased expression of MEK1 in HCC tumor cells lead to more growth in vivo and resistance to apoptosis in response to MEK inhibitor U0126 [252]. This study and others have also found increased ERK activation, which mediates upregulation of important factors in cell growth and proliferation and correlated with tumor size [252,253,254]. 

Pancreatic adenocarcinoma (PAC) is classified as a malignant neoplasm of the ductal or acinar cells in the pancreas and is generally diagnosed through endoscopic ultrasound and/or other imaging modalities [255]. With surgical and adjuvant therapy resulting in very low survival rates, alternative forms of treatment are required to produce better prognoses (Table 1). Mutations of the KRas gene are very common in PAC with a frequency of over 90% reported in cancer cells and are associated with a poor prognosis [256]. KRas has been shown to be required for sustained tumorigenic growth in advanced PAC, with loss of KRas expression leading to tumor regression [257]. KRas-induced activation of ERK, the main effector of the MAPK-ERK pathway, is thought to be responsible for a host of tumorigenic properties including tumor cell chemoresistance, invasion of pancreatic tumors, and the proliferation of pancreatic tumor cells [256]. KRas has been shown to be required for sustained tumorigenic growth in advanced pancreatic carcinoma, with loss of KRas expression leading to tumor regression [257]. In addition, KRas works with a series of other pathways to induce transformation, evade cell death or suppression of growth, and other malignant processes as reviewed in [256]. Although KRas mutations have been shown to be sufficient in transforming pancreatic cells into premalignant cell lines that can transition into PAC, there are patients who harbor KRas mutations displaying premalignant cell lines that never develop PAC. This indicates that although KRas is important in driving initial stages in PAC, other mutations may be needed in concert to develop PAC [258]. CRaf is important for both the initiation of KRas-driven PAC and progression, while BRaf is seemingly only required for late-stage PAC progression [259]. 

Non-small cell lung cancer (NSCLC) makes up 85% of lung cancer cases and comprises three main types of lung cancer: adenocarcinoma, squamous cell carcinoma, and large cell carcinoma. Treatment generally involves surgical resection with or without adjuvant therapy, however most diagnoses of NSCLC are made after metastasis where 5-year survival rates are approximately 6%, indicating a need for an alternative form of treatment [260]. KRas mutations and elevated KRas expression, as mentioned previously, are associated with driving forward metabolic activity associated with tumors (such as increased reliance on glucose, increased energy needs, increased TCA cycle activity, etc.) and previous research has shown similarities in metabolic activities between lung adenocarcinoma mouse models and human NSCLC tumor cells [261,262]. *KRas* mutation frequency and type can vary with lifestyle habits such as smoking [263]. The G12C mutation is associated with smoking tobacco use in patients with NSCLC [263]. Additionally, non-smokers and light smokers show fewer KRas mutations than heavy smokers and show a higher rate of the G12D mutation than the G12C mutation commonly seen in smokers and NSCLC generally [264]. There are conflicting reports concerning whether KRas mutations, including subtype (G12C, G12D, etc.) influence the survival outcome of NSCLC patients. Some reports have KRas mutations associated with poorer outcomes in NSCLC patients, especially those in early stages, going as far as finding that NSCLC patients with G12C and G12V KRas mutants had significantly reduced progression-free survival compared with those with G12D mutations or WT KRas [265,266]. Other reports, however, find that mutations, including those different in subtype, have no significant survival difference including during adjuvant chemotherapy [266,267,268]. 

In NSCLC and colorectal cancers (CRC), the KRas mutation has been linked to an increased expression in PD-L1, an immune checkpoint protein expressed in tumor cells that interacts with the PL1 receptor on T-cells as a method of avoiding immune-mediated cell death [269,270]. The MAPK-ERK pathway is known to both directly and indirectly result in the phosphorylation and inhibition of tristetraprolin, a protein that binds PD-L1 mRNA leading to its degradation [269]. An additional method by which the MAPK-ERK pathway mediates immune subversion is through the ability of KRas mutant tumor cells to generate suppressive T regulatory cells by secreting IL10 and TGFβ1 [271]. Modulation of antitumor immune responses occurs in other cancers with mutations in the MAPK-ERK pathway. Most notably, in BRaf mutant cancers like melanoma or CRC, immunosuppressive cytokines are upregulated inhibiting the release of inflammatory cytokines by dendritic cells and recruiting cells that downregulate antitumor responses [272]. Cancer-associated fibroblasts are among the cells mediating immunosuppression, and in BRaf mutant melanoma they have been found to upregulate PD-L1 aiding in immune evasion [273]. BRaf mutations have also been shown to reduce CD8+ T-cell tumor recognition and induce internalization of major-histocompatibility complex molecules in melanoma tumor cells [274,275]. Therefore, in patients with KRas and BRaf mutant cancers, immune checkpoint blockade has presented as an additional avenue of treatment to consider and has even produced positive results in patients with these mutations [276,277].

BRaf mutations are found in a smaller subset of NSCLC patients with the majority harboring V600E mutations and in adenocarcinomas [278]. In NSCLC mouse models, it has been found by various studies that CRaf was required for proper tumor initiation but not BRaf, as total ablation of BRaf but not CRaf allowed oncogenesis in KRas G12D and G12V mutant mice [279,280]. Additionally, depletion of CRaf but not BRaf in KRas mutant mice resulted in an inhibition of downstream ERK phosphorylation, an additional finding emphasizing the role of CRaf in KRas-driven cancer [281]. 

## 5. Targeting of Ras-Raf Signaling in Cancers

Exploring the role of MAPK signaling in the development of various cancers is vital in identifying potential therapeutic targets. Several MAPK signaling inhibitors relevant to cancer therapy have been compiled in Table 3. Upstream of Ras, SOS is a novel target for inhibiting the Ras protein. Several inhibitors have been manufactured including BI-3406 and BI 1701963 which bind to SOS and disrupt its interaction with Ras [282]. Preclinical research has shown that co-delivering SOS and MEK inhibitors counteracts the MEK resistance that is commonly seen after prolonged delivery of MEK inhibitors. There are multiple clinical trials testing BI 1701936 in solid cancer patients with and without KRas mutations. These trials involve the use of BI 1701963 in combination with different MAP/ERK protein inhibitors including KRas and MEK inhibitors (NCT04111458; NCT04975256; NCT04835714). Another novel target upstream of Ras is SHP2. Multiple phase I clinical trials using SHP2 inhibitors RMC-4630 and TNO155 are underway targeting KRas mutant NSCLC and other forms of cancer such as head and neck carcinoma (NCT04000529; NCT04330664; NCT03634982) [282]. Phase I clinical trials show that SHP2 inhibitors are tolerated well and reduced tumor volume in a subset of KRas G12C mutant NSCLC when combined with KRas inhibition [283]. Additional trials are being undertaken combining SHP2 inhibitors with other MAPK protein inhibitors, including the MEK inhibitor cobimetinib (NCT03989115; NCT03634982) [283]. SHP2 inhibitor BBP-398 is also being studied as a monotherapy in patients with advanced solid cancer without the BRaf V600E mutation (NCT04528836).

Ras itself has been considered as a target for cancer therapy. However, due to the difficulty in targeting Ras, contemporary efforts focused on targeting other proteins in the MAPK-ERK pathway despite Ras, particularly KRas, having high rates of oncogenic mutations in multiple cancers, especially PAC [259]. Indirect methods of targeting Ras include the use of farnesyltransferase inhibitors, which inhibit proteins that result in Ras’s localization to the cell membrane. Tipifarnib has shown efficacy in multiple phase II trials for acute myelogenous leukemia and myelodysplastic disorders but has not shown success in targeting advanced PAC [284,285]. Tipifarnib has also recently been designated as a breakthrough therapy by the FDA for HRas mutant head and neck squamous cell carcinoma after positive results from a phase II clinical trial and has shown preclinical activity in HRas mutant thyroid cancer cell lines [286,287]. Additional clinical trials are underway using tipifarnib for HRas mutant NSCLC and head and neck squamous cell carcinoma (NCT03496766; NCT04997902). While significant challenges arise in targeting specific KRas oncoproteins, breakthroughs in targeting the KRas G12C mutant have yielded the approval of Sotorasib as treatment for NSCLC [40]. These drugs represent the newest attempt at targeting the MAPK-ERK pathway, and approval of Sotorasib marks the first successful case of covalent Ras inhibition [38]. Although this new avenue of therapeutics presents with much optimism, KRas G12C inhibitor resistance has already been seen in preclinical models [38,39,40,41]. Genetically heterogenous tumors with mutations untargetable by G12C-specific drugs will inevitably develop resistance once the selective pressure of a drug treatment is exerted. To exacerbate the issue of resistance, tumors which lack dependency on KRas signaling may have intrinsic resistance, demonstrated by continued viability of tumor cells despite complete ablation of KRas signaling [288]. Researchers have explored additional explanations and have found that the mesenchymal cancer cell phenotype, which has been previously associated with a lower reliance on KRas for tumorigenic processes, instead relies on PI3K signaling, mediating resistance to G12C inhibitors [41]. These inhibitors are also specific for the G12C mutation which limits their use as this mutation is not common in several cancers including PAC (~1%), where the G12D and G12V mutations are markedly more common [289]. In accordance with discovery of the G12C inhibitor, there have been recent successful attempts at developing a rudimentary KRas G12D inhibitor that showed efficacy in cell-based assays and may point toward an additional method of treatment for KRas-driven cancers in the future [290]. 

Looking beyond targeting Ras directly, BRaf inhibitors developed to target the popular V600E mutant (vemurafenib, dabrafenib, etc.) have been pursued for their disruption of the MAPK-ERK pathway in cancers with BRaf-driven cancers, including BRaf mutant melanoma and NSCLC [193]. These inhibitors, however, have been ineffective in KRas- driven cancer [259,291,292]. An issue that can arise with use of Raf inhibitors alone is paradoxical activation. This was seen clinically when a patient with KRas mutant NSCLC was treated with vemurafenib and showed signs of a tumor flare indicative of paradoxical activation that may occur when weakly inhibiting Raf kinases [293]. Raf inhibitors were developed to avoid paradoxical activation by also targeting Raf dimers (e.g., LXH254, LY3009120, PLX8394, etc.) [259]. These have resulted either in high toxicity or were unable to inhibit CRaf, the particular Raf isoform important in the initiation of PAC [259]. Pan-Raf inhibitors are currently undergoing investigation for various solid tumors, aiming to avoid paradoxical activation. Sorafenib, a potent oral inhibitor of CRaf and BRaf, as well as several tyrosine kinases have been approved for advanced HCC [247,250,252,294,295,296]. There are several issues with the use of sorafenib as a monotherapy for advanced HCC. HCC commonly does not respond to sorafenib due to the heterogeneity of HCC cells [297,298,299]. When HCC does respond, the clinical benefit of the drug is limited to stabilizing HCC and resistance is also commonly seen after 6 months of treatment [297,298,299]. Synergistic regimen featuring sorafenib codelivery with many therapeutic drugs that are not MAPK/ERK inhibitors have been investigated for advanced HCC and include Artesunate, an antimalarial drug that suppresses angiogenesis and cell proliferation in HCC cell lines [300]. Artesunate is responsible for the creation of reactive oxygen species that contribute to increased apoptosis and, in the process, create phosphorylated ERK and STAT3 that concomitant sorafenib delivery reduces greatly, ultimately leading to reduced tumor growth [300]. Alternatively, sorafenib has been used alongside various compounds that inhibit the PI3K-AKT-mTOR pathway [299,301,302].

Downstream of Raf, MEK inhibitors have been developed to impede the MAPK-ERK pathway. Although effective for melanoma alongside codelivery of BRaf inhibitors, MEK inhibitors have generally also failed to treat PAC despite preclinical successes [259]. MEK inhibition has been hampered by two main issues, toxicity, specifically ocular toxicity, and resistance [258]. Drug resistance to MEK inhibitors that develops after prolonged use is thought to limit its effectiveness [303]. Mutations increasing MEK1 activation and bolstering resistance to MEK inhibitors have been shown to develop in colon cancer cell lines and even in a patient with resistant melanoma that developed after MEK inhibitor use [303]. Intratumor genetic heterogeneity has also been shown to develop in response to MEK inhibitor delivery in pancreatic cancer cell lines and may contribute to the lackluster results of MEK inhibitor use in clinical trials for patients with advanced PAC [304]. In addition, efforts to inhibit MEK have notably led to the removal of negative feedback against upstream receptor tyrosine kinases, resulting in continued Ras signaling despite MEK inhibition that may also contribute to resistance [305]. This issue is not isolated to MEK inhibitors as similar treatment evasion has been reported for G12C inhibitors, with tyrosine kinase blockade overcoming newly developed resistance, hinting that a potentially similar mechanism of evasion may exist for G12C inhibitors [288]. Raf inhibitors developed to avoid paradoxical activation by also targeting Raf dimers (e.g., LXH254, LY3009120, PLX8394, etc.) have resulted either in high toxicity or a lack of inhibition of CRaf, the particular Raf isoform important in the initiation of PAC [259].

Following MEK, ERK1/2 is thought to contribute to resistance to Raf and MEK inhibitors through loss of ERK feedback inhibition and subsequent ERK reactivation [306]. ERK1/2 feedback inhibition occurs via phosphorylation of proline rich regions on MEK that reduce MEK1 activity and interrupt activating phosphorylation and protein binding for MEK1/2 [307]. In addition, phosphorylated ERK levels have been associated with worse outcomes in patients with PAC [308]. Looking at preclinical models where resistance was developed in response to BRaf or MEK inhibitors, ERK inhibition was shown to be effective against resistant tumor cells [309,310]. Targeting PI3K and/or mTOR effector proteins downstream of KRas, has been an alternative route apart from targeting the MAPK-ERK protein pathway that is synergistic with combined MAPK/ERK inhibition and similar to ERK inhibition and has overcome resistant cancer cell lines [311,312]. Additionally, immune checkpoint blockade via the inhibition of PD1, a protein expressed on T-cells that can be manipulated by tumor cells in order to avoid immune activity, has also led to reduced resistance to MEK inhibitor trametinib and there are currently multiple Phase I and II trials combining MAPK/ERK inhibitors with PD1/PD1L inhibitors in patients with KRas G12C NSCLC (NCT03600883; NCT03600701; NCT02902029) [270]. The use of autophagy inhibitors alongside ERK inhibitors, as in the case of combining MEK and PDEδ inhibitors, has also shown enhanced antitumorigenic activity [308,312,313,314,315]. ERK inhibition, shown to upregulate PAC tumor cell reliance on autophagy and downregulate dependence on other metabolic processes, worked synergistically with hydroxychloroquine to inhibit growth in preclinical models of PAC [313]. ERK reactivation is often seen in EGFR inhibitor resistant NSCLC tumors, and therefore ERK1/2 inhibitors can be used as a part of a multidrug regimen to improve the effectiveness of these inhibitors representing another innovative use for ERK inhibitors [316,317]. There are concerns about the efficiency of delivering ERK inhibitors due to possible off-target toxicities and solubility problems, similar roadblocks shared by MEK inhibitors [318]. To solve these issues, there are efforts to deliver SCH779284 and other ERK inhibitors alongside standard chemotherapeutics more efficiently using nanoparticles [318].

## 6. Conclusions and Future Perspectives

The Ras-Raf-MEK-ERK pathway is a critical component of cell cycle control, exerting strict regulation over proliferation and differentiation. Mutations within this highly conserved signaling pathway have proven to be key drivers of numerous human blood and solid cancers. As key regulatory points within the MAPK pathway, Ras and Raf exist as multiple isoforms with different characteristics regarding activity and involvement in oncogenesis. Thus, targeting Ras signaling has been a topic of discussion and research. Most notably, a recent breakthrough in the approval of Sotorasib as a KRas G12C inhibitor has ignited hope in what was previously considered an undruggable target. In addition, there are a plethora other KRas G12C inhibitors in clinical trials for NSCLC and CRC such as JAB-21822, GFH925, LY3537982, and most notably MRTX849 which is leading with phase III trials (Table 3). Other novel targeting involving the MAPK-ERK pathway include SOS inhibitors, particularly BI 1701963 which is undergoing phase I/II clinical trials, and Shp2 inhibitors (Table 3). However, challenges remain in mutational subgroup specificity leading to drug resistance by tumor genetic heterogeneity, and a need for pan-inhibitors remains. Furthermore, downstream challenges may arise in paradoxical activation by MAPK/ERK inhibitors, highlighting the necessity of continued research to circumvent these challenges.

Alternatively, nutraceuticals are under-researched and interact with MAPK pathway. Silibinin and Curcumin have been used with sorafenib and have interacted synergistically to inhibit tumor growth in HCC preclinical models [319,320]. Silibinin as a monotherapy has inhibited tumor cell proliferation, metastatic potential, ERK1/2 phosphorylation, and levels of downstream cyclin proteins [319,321,322,323]. Meanwhile, Curcumin, one of many plant-derived polyphenols, has been investigated for use in multiple cancers and pathologies, including HBV-induced HCC, due to its antioxidant, antiviral, and anticancer functions [324,325]. Further research is needed to study the use of plant-derived compounds for use with and without other MAPK/ERK inhibitors. Other novel methods of targeting the Ras-Raf pathway include chimeric antigen receptor T-cell therapy, which involves the use of modified T-cells that recognize antigens specific to KRas mutant cancer cells and induce T-cell response independent of the usual processing and presentation by antigen presenting cells [326]. There is currently an active phase I/II clinical trial using G12V-specific T-cell therapy for pancreatic cancer (NCT04146298). Additional methods using the body’s immune system include peptide vaccines that work by inducing an immune response to a synthetic peptide associated with tumor cells that are processed and presented by class I and class II major histocompatibility complex molecules. There are two phase I clinical trials using KRas-targeted peptide vaccines: one prophylactically for patients at risk of developing pancreatic cancer and the other in patients with resected microsatellite stable pancreatic or colorectal cancer (NCT05013216; NCT04117087). Lastly, vaccines using mRNA instead of peptides are being adopted for use against cancer and have a series of improvements over peptide vaccines including the ability to encode entire tumor antigens, resulting in greater epitope presentation to T-cells and stimulating a larger T-cell response [327]. The mRNA vaccine mRNA-5671 targets mutant KRas tumor cells and is in a phase I clinical trial for patients with advanced or metastatic NSCLC, PAC, and CRC (NCT03948763).

Furthermore, there are certain types of cancers that have not been thoroughly investigated in regards to the role played by the MAPK/ERK pathway such as acute myeloid leukemia (AML) and acute lymphoblastic leukemia (ALL), hematologic malignancies that are differentiated by clonal proliferation of either myeloid or lymphocytic cells, respectively. More work is needed to elucidate the involvement of Ras signaling in AML and ALL. The same is true for HPV infection and its associated cervical, oral, anal, and other cancers. Similarly, while the involvement of the Ras-Raf pathway is increasingly understood in ATLL and BL, no treatments targeting it exist for these cancers. While much progress has been made in targeting MAPK/ERK signaling and the growing body of knowledge surrounding Ras-Raf involvement in oncogenesis yields great potential, substantial efforts must be made to translate these targets into safe, efficacious treatment for a wide variety of cancers.

## Figures and Tables

**Table 1:** Various blood cancers with Ras-Raf mutations.

Blood Cancer	Ras-Raf Pathology	General Treatment Protocol	5-Year Survival	References
Myelodysplastic Syndromes	NRas, KRas mutation in 7–48% of patients.	Use of erythropoietin stimulating agents to mitigate symptoms. Allogeneic stem cell transplant for higher risk patients.	29%	[163,164,165,166,167]
Acute Myeloid Leukemia	NRas, KRas mutations in 10–27% of de novo patients.	Induction via cytarabine and the addition of an anthracycline for patients followed by consolidation.	24%	[167,168,169,170]
Acute Lymphoblastic Leukemia	NRas, KRas mutations in 5–22% of patients. BRaf mutations have been found in infants and children with acute B and T-cell lymphoblastic leukemia. Small sample found ~21% of ALL patients with BRaf mutations.	Induction using vincristine, corticosteroids, L asparaginase, and an anthracycline for patients followed by consolidation.	Between 30 and 45%	[171,172,173,174]
Chronic Myelomonocytic Leukemia	NRas, KRas mutations in 30–50% with CMML. Subset of patients with CMML-1 presented BRaf mutations (~7%).	Lack of consensus about a treatment that markedly expands overall survival rate. Hypomethylating agents have shown some promise and are approved by the FDA for CMML. Allogeneic stem cell transplantation regarded as only curative treatment.	18.5%	[175,176,177,178,179]
Chronic Myeloid Leukemia	NRas mutations in up to 1/3 of atypical CML. Chronic CML patients present up to 17% of Ras mutations, up to 58% of acute CML patients have Ras mutations.	Standard treatment involves the use of tyrosine kinase inhibitors in sequence.	61%	[166,169,180,181]

**Table 2:** Various solid cancers associated with Ras-Raf mutations.

Solid Cancer	Ras-Raf Pathology	General Treatment Protocol	5-Year Survival Rate	References
Pancreatic Adenocarcinoma	KRas mutations in up to 90% of patients. BRaf mutation in 14% of patients.	Surgical resection and/or chemotherapy.	less than 5%.	[166,182,183,184,185]
Melanoma	Ras mutations in up to 36% of patients. BRaf mutations in 27–70% of patients.	Surgical resection. Chemotherapy and novel targeted therapies, including BRaf inhibitors, may be used when surgical resection is not possible.	91%.	[169,186,187,188,189]
Non-Small Cell Lung Cancer	KRas mutations in 22–36% of patients. BRaf in ~2 to 5% of patients.	Surgical resection and/or chemotherapy.	25%	[190,191,192,193,194,195]
Colorectal Cancer	KRas mutations in 40–60% of patients. BRaf mutation in 18% of patients.	Surgical resection and/or chemotherapy.	65%	[169,187,196,197]
Seminoma	KRas, NRas mutations in 7–40% of patients.	Radical orchiectomy with subsequent chemotherapy.	86.4%	[166,198,199,200]
Bladder Cancer	HRas, NRas mutations shown in up to 80% of patients, although Ras mutations generally considered present in 10% of patients.	Immunotherapy and/or chemotherapy, with radical cystectomy after invasion of muscle.	80.8%	[201,202,203,204]
Hepatocellular Carcinoma	NRas mutations in 30%, KRas mutations in 1.6% of patients. BRaf mutations in 14% of patients.	Surgical resection, liver transplantation, and/or chemotherapy.	15%	[156,166,187,205,206]
Ovarian Cancer	KRas mutation in 13.7% of patients. BRaf mutations in 4–30% of patients	Cytoreductive surgery followed by chemotherapy.	40%	[187,188,207,208]
Renal Cell Carcinoma	Ras mutations in up to 13% of patients.	Tyrosine kinase, m-TOR, and VEGF inhibitors. Other targeting therapies including immunotherapy are used as well.	Between 92.5 and 12% depending on localization.	[166,209,210,211]

**Table 3:** Inhibitors of MAPK cascade in active clinical trials.

Inhibitor	Notes	Clinical Trials	Targeted Cancers
BI 1701963	Binds SOS1 protein, inhibiting its activation of KRas	NCT04111458, NCT04975256, NCT04835714	Lung, colon, and lung cancer
RMC-4630	Selective inhibitor of Shp2, indirectly inhibiting KRas	NCT03634982, NCT03989115, NCT04916236	Pancreatic, colorectal, and non-small cell lung cancer
TNO155	Selective inhibitor of Shp2, indirectly inhibiting KRas	NCT04330664, NCT04292119, NCT04000529	Lung, head and neck, esophageal, gastrointestinal and colorectal cancer
BBP-398	Selective inhibitor of Shp2, indirectly inhibiting KRas	NCT04528836	Advanced solid tumors
Sorafenib	Inhibitor of multiple intracellular and cell surface kinases such as CRaf and BRaf that are involved in tumor cell signaling, angiogenesis, and apoptosis	NCT01730937, NCT03518502, NCT01371981	Liver and thyroid cancer, leukemias
Sotorasib (AMG 510)	KRas inhibition specific to G12C mutation	NCT03600883, NCT04303780, NCT04933695	Non-small cell lung cancer
MRTX849	KRas inhibition selective to the G12C mutation	NCT04793958, NCT04685135	Non-small cell lung and colorectal cancer
JAB-21822	KRas inhibition selective to the G12C mutation	NCT05009329, NCT05002270	Non-small cell lung and colorectal cancer
GFH925	KRas inhibition selective to the G12C mutation	NCT05005234	Advanced solid tumors
LY3537982	KRas inhibition selective to the G12C mutation	NCT04956640	Non-small cell lung, colorectal, endometrial, ovarian, and pancreatic cancer
Tipifarnib (R115777)	Farnesyltransferase inhibitor that prevents post-translational processing of Ras proteins. Prevents Ras from membrane binding	NCT03496766, NCT04997902, NCT03155620	Non-small cell lung and head and neck cancer, non-Hodgkin lymphoma
Rigosertib	Targets mutated Ras pathway in leukemia by interacting with effectors proteins containing Ras binding domains	NCT04263090, NCT04263090, NCT03786237	Myelodysplastic syndrome, non-small cell lung cancer, and squamous cell carcinoma
Trametinib (GSK1120212)	Non-ATP-competitive inhibitor of MEK1/2. FDA approved, suggested to use in combination with BRaf inhibitors due to resistance.	NCT03340506, NCT04940052, NCT02101788	Non-small cell lung, thyroid, ovarian and peritoneal cancer, melanoma
Mirdametinib (PD0325901)	MEK1/2 inhibitor (derivative of CI-1040)	NCT02022982, NCT03905148, NCT04923126	Ovarian, endometrial, pancreatic, thyroid, and non-small cell lung cancer, melanoma, glioma
Selumetinib (AZD6244)	Highly selective, non-ATP-competitive MEK1 inhibitor.	NCT04576117, NCT03705507, NCT03705507	Non-small cell lung cancer, glioma, leukemia
Binimetinib (MEKTOVI; MEK162)	Highly selective, non-ATP-competitive MEK1/2 inhibitor.	NCT04657991, NCT02928224, NCT03843775	Colorectal cancer, melanoma, and other *BRaf* mutant malignancies
RO5126766 (CH5127566)	Selective Raf/MEK1/2 inhibitor	NCT02407509, NCT03875820, NCT04720417	Non-small cell lung, ovarian, and colorectal cancer, multiple myeloma, melanoma
HL-085	Selective MEK1 inhibitor	NCT03973151, NCT03990077, NCT03781219	Non-small cell lung cancer, melanoma, and *BRaf* mutant solid cancers
Ulixertinib (BVD-523)	ATP-competitive, reversible inhibitor of ERK1/2	NCT04145297, NCT03417739, NCT04488003	Gastrointestinal cancers, melanoma, and *BRaf* mutant solid cancer

## Abbreviations

MAPK	Mitogen-activated protein kinase
ATLL	Adult T-cell leukemia/lymphoma
HTLV-1	Human T-cell lymphotropic virus type 1
ERK	Extracellular signal-regulated kinase
JNK	c-Jun N-terminal kinase
EBV	Epstein–Barr virus
HBV	Hepatitis B virus
HCV	Hepatitis C virus
HPV	Human papillomaviruses
GDP	Guanosine diphosphate
GTP	Guanosine triphosphate
Ras-GRF	Ras protein-specific guanine nucleotide releasing factor 1
FTase	Farnesyl transferase
GGTase1	Geranylgeranyl transferase 1
Rce1	Ras converting enzyme
Icmt	Isoprenylcysteine carboxyl methyltransferase
PDEδ	Phosphodiesterase delta
GPCR	G-protein-coupled receptors
IL	Interleukin
JAK2	Janus kinase 2
SH2	Src homology 2 domain
Grb2	Growth factor receptor bound protein 2
SOS	Son of sevenless
GEF	Guanine nucleotide exchange factor
Gal-1	Galectin-1
EGFR	Epidermal growth factor receptor
PKA	Protein kinase A
PLCβ	Phospholipase C-β
RBD	Ras-binding domain
PAK	p21-activated protein kinase
PKC	Protein kinase C
BL	Burkitt’s lymphoma
NHL	non-Hodgkin’s lymphoma
HCC	Hepatocellular carcinoma
NSCLC	Non-small cell lung cancer
PAC	Pancreatic adenocarcinoma
CRC	Colorectal cancer
Shrp2	Src homology region 2 domain-containing phosphatase 2
Csk	C-terminal Src kinase
SFKs	Src family kinases (SFKs)
Epac-1	Exchange protein directly activated by cAMP
PLCγ	Phospholipase C-γ
PAG	Phosphoprotein associated with glycosphingolipid-enriched microdomains
PP1	Protein phosphatase 1
